# The Needs Of Canadian Women Veterans Experiencing Homelessness: A National Study Seeking Functional-Zero Homelessness

**DOI:** 10.1192/j.eurpsy.2026.10188

**Published:** 2026-07-31

**Authors:** C. Forchuk, R. Booth, A. Rudnick, A. Nazarov, D. Richardson, J. Serrato

**Affiliations:** 1 Western University; 2 Mental Health Nursing Research Alliance, Lawson Research Institute, London; 3 Dalhousie University, Halifax; 4 Nova Scotia Health Authority, Dartmouth; 5 St. Joseph’s Health Care, London, Canada

## Abstract

**Introduction:**

Pathways to homelessness for Canadian Veterans are complex and influenced by pre-existing health conditions and experiences related to military service. Conservative estimates suggest there are 2,400 Canadian Veterans experiencing homelessness with 30% identifying as women. Although 52 studies have examined homelessness among Women Veterans, none have been conducted outside the US.

**Objectives:**

The objectives of this national study are twofold: (1) to co-create solutions to homelessness among Canadian Women Veterans; and, (2) to address the knowledge gaps in the literature. This study aimed to deepen the understanding of how gender intersects with psychosocial factors; explore the service barriers faced by Women Veterans; and generate strategies to reduce their risk of homelessness.

**Methods:**

While recruitment is currently ongoing, up to 160 Women Veterans with current or previous experience of homelessness will participate in individual, semi-structured interviews that combine open ended questions with a brief set of closed-ended items. The qualitative component is gathering data regarding lived experiences and suggestions for an ideal program to support Women Veterans. A thematic analysis informed by ethnography is being utilized for qualitative analyses to ascertain cultural intersections within this unique population. Quantitative data includes demographics, housing history and quality of life.

**Results:**

This study aims to deepen understandings of how gender intersects with psychosocial factors, explore the service barriers faced by Women Veterans, and generate strategies to reduce their risk of homelessness. Preliminary emergent findings uncovered the following concepts: (1) trauma and mental health challenges are widespread, requiring additional support; (2) there is a need for greater awareness of supportive Veteran services; (3) transitional housing often requires living alongside men; and, (4) there is a need for gender-specific housing and outreach services. Perceptions of sexism were also widely reported. When asked what an ideal program would be, the participants largely suggested the following: a) Housing specifically for women Veterans only; b) greater outreach to find women Veterans; c) peer support d) financial and employment support; e) more mental health supports made more available and accessible.

**Conclusions:**

The preliminary findings revealed that more mental health supports and gender-specific models need to be developed. This data and analyses generated from this study will contribute a significant step towards laying the foundations for future Veteran research across Canada and the international community. It is hoped that this study will accelerate population-level functional zero homelessness in communities across all of Canada through the development of gender-specific recommendations for practice, policy and housing.

**Disclosure of Interest:**

None Declared

